# Transformative learning in the setting of religious healers: A case study of consultative mental health workshops with religious healers, Ethiopia

**DOI:** 10.3389/fpsyt.2022.897833

**Published:** 2022-09-13

**Authors:** Yonas Baheretibeb, Sophie Soklaridis, Dawit Wondimagegn, Maria Athina (Tina) Martimianakis, Samuel Law

**Affiliations:** ^1^Department of Psychiatry, School of Medicine, College of Health Sciences, Addis Ababa University, Addis Ababa, Ethiopia; ^2^The Centre for Addiction and Mental Health, University of Toronto, Toronto, ON, Canada; ^3^Department of Paediatrics, Temerty Faculty of Medicine, University of Toronto, Toronto, ON, Canada; ^4^Department of Psychiatry, Temerty Faculty of Medicine, University of Toronto, Toronto, ON, Canada

**Keywords:** transformative learning, traditional healers, collaboration, psychiatric practitioners, Holy water

## Abstract

**Objective:**

Psychiatric interventions that consider the socio-cultural and spiritual traditions of patients are needed to address stigma and improve access to mental health services. Productive collaboration between traditional healers and biomedical practitioners hold promise in such efforts, and applying tenets of transformative learning hold potential for mitigating an overemphasis on biomedical models in such collaboration. We present a framework for how to engage in health system reform to enhance mental health services in communities that are distrustful of, or unfamiliar with biomedical approaches. Our research question was how to bridge two seemingly opposing paradigms of mental health care, and we sought to understand how the theory of transformational learning (TLT) can be applied to learning among Religious healers and biomedical practitioners in culturally appropriate ways to improve collaboration.

**Methods:**

TLT informed the development, implementation, and evaluation of an educational intervention in Addis Ababa, Ethiopia that aimed to improve delivery of mental health services at two Holy water sites. The initiative involved both psychiatrists and religious healers with extensive experience providing care to mentally ill patients. Using a focused ethnographic approach that incorporates document analysis methodology, this qualitative study examined recordings and minutes of stakeholder meetings, workshops and informal interviews with participants, analyzed for evidence of Mezirow's 11 stages of transformative learning. A participatory action approach was used to encourage practice change.

**Results:**

All participants exhibited a high degree of engagement with the of the collaborative project and described experiencing “disorienting dilemmas” by Mezirow's classic description. Opportunities to reflect separately and in large groups encouraged a re-examination of attitudes previously contributing to siloed approaches to care and led to instrumental changes in mental health care delivery and a higher degree of coordination and collaboration between psychiatrists and traditional healers.

**Conclusion:**

Our study demonstrates the utility of TLT in both the design and evaluation of initiatives aiming to bridge cross-cultural and cross-professional divides. The learning process was further enhanced by a collaborative participatory action model adjusted to accommodate Ethiopian socio-political and cultural relations.

## Introduction

Health care approaches that are attentive to the socio-cultural and spiritual traditions of patients are needed to address the ubiquitous stigma against mental illness, and improve acceptance and access to mental health services. Interventions applying tenets of transformative learning hold potential to improve these aspects. In 2010, the Lancet Commission called for a major institutional and instructional reform in health education to meet the demands of the new era where information technology, global movement, increasing inequality within and between countries, and gaps between pedagogical theory and practices have brought new challenges to health care (1). The Commission articulated its vision as follows: “all health professionals in all countries should be educated to mobilize knowledge and to engage in critical reasoning and ethical conduct so that they are competent to participate in patient-centered and population-centered health systems as members of locally responsive and globally connected teams” (1). For this vision and process, the commission proposed reforms aimed at making “transformative learning” the highest-level of learning outcomes.

Transformative learning involves changing existing beliefs and thought patterns through the use of discourse and critical reflection (2, 3). Ideally, the learner develops an open and accommodating view of the topic. Beyond transmitting content, transformative learning requires skills for ongoing reflection and meaning making (4). Transformative learning can involve any combination of spiritual, political, emancipatory, or developmental components (5–8), and can occur in both formal and informal learning environments that are facilitated by educators or be self-directed (9, 10). The goal of this kind of learning is to move beyond transmission of instrumental knowledge by awakening the learner to a new manner of viewing and examining the world (6, 11).

Transformative learning also allows participants to engage in knowledge construction by applying new information and broadening existing meaning-making schemes (6). According to Mezirow's transformational learning theory (TLT), this is accomplished through an eleven-phase process: a disorienting dilemma, self-examination, critical assessment of assumptions, recognition of discontent and identification with similar others, exploration of new options, planning, acquiring knowledge for plans, experimenting with new roles, building confidence, reintegration, and renegotiation of relationships (2, 11). As the learner moves through these phases, critical reflection supports the consideration of new concepts and information. Different pedagogical approaches can help learners consider how to use and apply the new information including reconstituting themselves differently as a social or professional actor (2).

Mezirow acknowledges that self-empowerment is limited by social, historical, and cultural conditions, and that “our life histories and language are bound up with those of others. It is within the context of these relationships, governed by existing and changing cultural paradigms, that we become the persons we are” (3). In other words, learning is influenced by the cultural, historical, and social elements of the context surrounding learners. However, Mezirow's theory stops short of elaborating on how this change, from the perspective of culture, happens. In fact, how cultural considerations affect transformative learning is an under-studied area (12).

Reassuringly, Mezirow's TLT, as a major theory of adult education, can be further adapted in its multi-phase process to address this need. TLT could facilitate ways to tap into marginalized cultural knowledge construction systems to stimulate personal change by encouraging a process of new meaning-making. An aspect of the TLT that is relevant to the proposed culture-sensitive transformation is what he calls the “sociolinguistic” meaning perspectives. These perspectives “are understood as habits of expectation assimilated primarily from one's culture and language” (13). Mezirow's theory also recognizes the important roles of both culture and context and the fact that there is an element of negotiated understanding that is connected or integral to learning and knowledge.

In Ethiopia, access to biomedically-based mental healthcare is very limited and mostly centralized in the capital city of Addis Ababa. The Amanuel Mental Specialized Hospital and a small number of outpatient clinics constitute the majority of services (14). Outside the city, there is a paucity of formal or semi-formal support from the government clinics, community health stations, or non-governmental organizations (NGOs) (15). Although mental health is being integrated into primary care in several pilot sites across Ethiopia, there exists a stark treatment gap (16). For example, in rural settings, only 58% of people with psychosis report lifetime access to psychiatric care (15). In Addis Ababa, only 10% of street homeless persons with psychosis have ever accessed psychiatric treatment (17). Given the resource limitations, the majority of the population in Ethiopia rely on traditional healers for all aspects of health care. Although traditional healing practices are complex and diverse across the different ethnic groups, most traditional healers are valued for their wisdom, knowledge, and their known ability to understand, link, and situate health problems within the health-seeker's social and cultural beliefs (18). Particularly in mental health, traditional and religious healing is the mainstay of such services, along with family members as the main care providers for people with mental illness (19). In Addis Ababa, for example, approximately half of the individuals who ultimately seek psychiatric care for mental disorders have previously attended traditional or religious healers (17).

One of the most common healing methods used by traditional or religious healing for mental illness in Ethiopia is Holy water (*tsebel*). Traditional and religious healers believe that Holy water has curative properties for mental as well as physical illness and minor social difficulties (17). Most Ethiopian Orthodox Christian churches have their own Holy water source and are considered by many to be reputable healing sites. Given its popularity, availability, and cultural acceptability, many non-Orthodox Christians, or Non-Christians also use Holy water as treatment (20, 21). Holy water is free to use and accessible to anyone. The process is usually guided or administered by Holy water priests, involving a short course of splashing, bathing or drinking the water, and attending prayers led by the priests (21). Those with more severe complaints, most commonly severe and chronic mental illness, may reside nearby a Holy water treatment site for months or years to receive on-going treatment.

Beyond its practical availability and ease of access, there is also increasing recognition of the value, efficacy, and advantages of many aspects of traditional healing (22–25). Yet, despite the important role traditional healing plays in the current mental health care, and often being a method of choice by the majority of patients and their families in settings like Ethiopia and many other parts of the world, its relationship with biomedical forms of care has historically been highly antagonistic. This state of relationship is most likely related to their widely different understandings of mental illness (26, 27), distrust, competition, and generally unfavorable viewing of each other, even if many biomedical practitioners grew up with traditional healing (28, 29). Given the reality that substantial advantages and usefulness exist in both systems, and the centrality of traditional healing in the health care of Ethiopians, there seems to be a unique and largely missed opportunity for collaboration between traditional healers and psychiatric care providers.

There are several compelling rationales for such collaboration in health system planning (25, 30, 31). First, such collaboration could increase overall service access and aid the early detection and treatment of mental illness, which may improve long-term outcomes (32). Second, it could contribute to minimizing harmful practices in some circumstances, such as giving up, neglecting, or forcefully restraining patients. It is estimated that traditional providers use physical restraint, particularly in the care of people with severe mental illness in 4% of cases in Kenya, 21% in Ghana and 63% in Nigeria (33). Third, it could raise awareness amongst psychiatric practitioners of the spiritual needs of people with mental disorders (34). Last but not least, combining traditional and biomedical healthcare could capitalize on the powerful influence of traditional practitioners to reduce stigma and encourage community support for people with mental illness (14).

Transformative learning could facilitate this important connection between biomedical practitioners and traditional healers. Changes that inspire collaboration occur through learning, and this case in point is deeply cross-cultural. Based on the concepts of TLT, the current study investigates how multiple consultative workshops between psychiatric service providers and religious healers transformed practice and world views across the two groups. The targeted participants for the current study are established professional healers from the two paradigms of care. As experienced and adult learner participants, the study's key transformative learning aspects focus on creating a disorienting dilemma regarding the value of the “other,” promoting self-examination and assessment of long and deeply identified assumptions, and exploration of new meaning and professional roles that could be utilized to support the general learning of new knowledge and reform of healthcare practices (4, 11, 35–38). To the best of our knowledge, application of TLT in entrenched and established populations such as religious healers' and psychiatric practitioners' education is relatively unknown, and no study has examined transformative learning among these two groups in an Ethiopian context.

## Methods

The main research question of the study was how to bridge two seemingly opposing paradigms of mental health care. We thus sought to understand how the concept of TLT can be applied to learning among psychiatrists and religious healers in culturally appropriate ways to improve collaboration. We carried out this qualitative study using a focused ethnographic approach (39), incorporating document analysis methodology to examine the transformative learning process and outcomes of participants (40). A focused ethnographic approach is well-suited for the study as it is a deeply descriptive and analytic project, using a variety of methods for data collection, capturing different forms of data over a discreet number of events. In addition, the research team had built a good relationship with the participants before the start, establishing a shared purpose and knowledge sharing approach. Through deliberate consideration of the many levels of clinical, social, cultural, and political contexts of the project, the research team engaged in ongoing reflection of how their respective lived experience impacted the interpretation of the findings. The research team had different cultural backgrounds and varied experienced with traditional and biomedical approaches to mental health. Participant feedback was used to ensure authentic representation of participant perspectives in the study (41). The study benefited from methodologies practiced in document analysis as we used a systematic procedure to analyze all available documentary evidence to answer our general research questions, with repeated reviews, examinations, and interpretations of the data to gain meaning and knowledge on the outcome of interest (40).

The study data were derived from engagements, multiple workshops, and informal interviews with religious healers. A research assistant with qualitative research skills, experience and knowledge of both religious healing and biomedical approaches took notes and memoranda, timed meetings, and collected all salient information related to meetings and workshops with religious leaders, religious healers, Holy water patient attendants, and biomedical mental health practitioners. The forms of data included transcriptions of recorded audio and videos of the consultative workshops, pre- and post-workshop assessment questionnaires, manuals, letters, charts, program proposal, and event programs. The study aimed for a collective perspective and data amongst the different participants were not segregated for sub-analysis. Participants in these workshops consented to have their deliberations and informal interviews used as part of this study.

We applied Mezirow's TLT as a theoretical framework to structure the current study (13). The research assistant and the first author (YB) carried out the initial data analysis, with further review of the analysis from the third co-author (DW) and an independent consultant with strong qualitative research skills. The document analysis aimed to measure transformative learning within the contexts of multiple workshops activities. As informed by the TLT framework, all collected data were examined and interpreted by the research team to give voice and meaning around the different stages of learning, and narrative plot elements that would indicate a causal sequence of events indicating potential transformation of the learners (40). Our data analysis was also informed by thematic analysis techniques (42). The thematic analysis of the documents involved skimming (superficial examination), closer reading (thorough examination), and iterative interpretation, making notes and memoranda at each stage to document findings, aiding communication among researchers, and continuous updating and informing the overall analysis. The research team was sensitized to all notions and themes of learning and change and focused on whether religious healers experienced attitudinal shifts or modified their Holy water treatment practices. Specifically, we analyzed the workshops' documents according to the eleven phases of transformation identified by Mezirow and the decision to use all content of the eleven phases was data-driven (43).

In addition to reflexive procedures adopted by the research team, the rigor of the study relied on a number of processes. First, the transcriptions of the recordings of the consultative workshops, where checked against the recordings to verify the accuracy of the transcription. Second, the first author (YB) consulted peers who were knowledgeable about the subject of the study to inform the methodology, and reviewed the data collected and analyzed by the research assistant to evaluate the consistency of the analysis. And lastly, a few of the study's participants had the opportunity to review and confirm that the interpretation and analyses of the data accurately reflected their contributions.

## Description of engagement meetings and workshops

### The initial engagement

The study took place in Entoto, which is an elevated area on the northern perimeter of Addis Ababa, Ethiopia. The two Orthodox Christian churches in Entoto, St. Mary's and St Michael's, are within 2 h walk of each other. These churches have popular Holy water sites and are known to specialize in treating mental illnesses (there are other sites that are more known for physical and infectious illnesses). Each church has several prayer compounds and separate buildings with piped Holy water available at various points. Ceremonies, involving prayer, baptism and drinking of Holy water are conducted daily by the priests. At any given time ~550 and 1,000 people live around St Mary's and St Michael's Holy water sites, respectively, in basic dormitory-style houses run by Holy water attendants.

To understand the religious healers' background, approaches in mental health care and to identify potential common goals, the Principal Investigator (PI) (YB) visited both churches. YB introduced himself as a psychiatrist at an University and a community organizer at the Ethiopian Mental Health Society—a local NGO that promotes mental health, and had an informal discussion with the priests there about mentally ill populations at the Holy water sites. The discussion mainly focused on common issues like stigma, alienation, and segregation of patients with mental illness from other patients. After the discussion, YB intuitively felt the interest to work together. To facilitate more acceptance and to respect the religious tradition, YB approached the headquarters of the Orthodox Church in the capital (Addis Ababa) that is overseeing all church functions in the country. Growing up with personal experience of traditional healing, and continued respect for the practice as a biomedical practitioner, YB introduced to the headquarter church leaders the idea of possible collaboration, and had long discussions on thorny, but potentially improvable issues such as physical abuse, stigma and unhygienic conditions faced by mentally ill people around the Holy water treatment sites. These uncomfortable topics and discussions for both parties created a situation where the church leaders learned that their general impression and beliefs about the Holy water site environments had not been entirely accurate—this fact became the key spark in a general desire to improve the conditions for the patients, and YB got a letter of support from headquarters that strongly recommended both the St. Mary's and St. Michael's churches to work with YB. YB believes that being both culturally and religiously sensitive, and the church officials' sympathy toward his inquiry helped in obtaining the approval. Since the Orthodox Christian Church is highly hierarchical, the letter from its headquarters proved to be useful as a means of introduction, and made the local priests practicing Holy water treatment more comfortable as the headquarters' authorization facilitated their engagement in such novel conversation with someone working outside their religious community.

There was a concern whether this approach may be too top-down; but, to his relief, YB was welcomed by the priests and the process was warm and cordial, his questions were taken seriously, and there were genuine interest and curiosity for a dialogue. With the rapport and confidence built from these initial meetings, YB shared the experience with the board of his NGO and the board decided to further engage the priests with a formal, information-sharing series of consultative workshops. The hope was that the consultative workshops could lead to a shared agenda for more collaboration. YB and a research assistant knowledgeable about Holy water treatment and qualitative studies had two brainstorming and preparatory meetings with two religious leaders to prepare for the workshops. In the meetings, the church leaders discussed the sensitivity and uniqueness of the planned consultative workshops with the Holy water priests. After the meeting YB identified the following principles to conduct the workshops: (1) Fostering and promoting trusting and supportive relationships– e.g., facilitating a climate that encourages expressions of different perspectives and opinions; (2) Promoting respectful self and collective reflections—e.g., through sharing of case scenarios; (3) Creating personal and meaningful experiences for both parties—e.g., through visiting patients at respective Holy water sites and psychiatric hospitals; (4) Communicating openness and readiness to collaborate—e.g., exploring models and options for such. Based on these principles, YB and his research assistant drafted the agenda of the consultative workshops and identified two co-leaders/facilitators (One priest healer and YB), invited participants and developed detailed activities for the workshops.

### The first consultative workshop- exploration of respective explanatory models

The first workshop was organized within the vicinity of St. Mary's church. YB and the church leaders agreed on a draft agenda prior to the consultative workshops as follows: Day one: Opening and introduction, concept of mental illness, religious view of mental illness, modern view of mental illness. Day two: Stigma, segregation and alienation of patients with mental illness, case report sharing and discussion of treatment using Holy water, case reports and discussions at a hospital.

A total of 55 participants attended the workshop. The participants included senior religious healers, senior clergymen, University mental health practitioners, representatives from the Ethiopian Mental Health Society and Holy water patients' attendants. Consistent with the local reality, there were 52 male participants, and 3 females who were from the NGO and the University. About half of the participants were between the ages of 30 and 60, with the other half aged between 61 and 70. About 20 participants were senior clergymen or senior religious healers and have completed the church school system “divisions,” which is one of the oldest educational systems in Ethiopia that teaches biblical, musical, philosophical, ethical and general knowledge. Five of the senior clergymen completed modern education in Ethiopian public high schools. All senior clergymen and religious healers had more than 10 years of experience of working with mentally ill people. Two participants were associate professors of psychiatry and had more than 15 years of experience working as psychiatrists. There were also two assistant professors of psychiatry with more than 5 years of work experience, and nine psychiatric nurses with five or more years of work experience.

In a show of respect to the church and its customary practices, YB invited church leaders from the capital Addis Ababa. The opening speech at the first consultative workshop was given by one of the influential elderly leaders of the church, who emphasized the importance of collaboration in fighting stigma related to mental illness. Following the opening speech, YB and the priest co-lead facilitated the workshop. In these discussions, the religious healers and Holy water attendants described, shared and examined their knowledge, assumptions, expectations and feelings regarding the causes, symptoms and treatment of mental illness. In most of the religious healers' comments, causes of mental illness were attributed to religious and spiritual sources such as witchcraft, curses, punishment from God and ancestors for sins and wrong doings.

Nevertheless, religious healers' casual explanations also included factors that were similar to those of the psychiatrists—these included excessive alcohol use, khat use, drug abuse, heredity, physical illnesses, injury and old age. The mental health practitioners shared with the religious healers their pleasant surprise from learning the mixed model of explanations as they did not expect this from the religious healers. After carefully listening to the religious healers, aided and organized by the Explanatory Model framework (44, 45), the psychiatrists in attendance shared the biomedical explanatory model of mental illness that also included how social, cultural and service contexts affect the cause and course of mental illness. Their report also created a sense of surprise for the religious healers, expecting these medical professionals to only cite biological causes. Another issue that created unexpected experiences for mental health professionals was the similarities in the process of making diagnosis by religious healers, which includes observing and taking a careful history from the patients, their family and attendants, observation before and during Holy water treatment rituals, and comparisons to known patients.

Subsequently the workshop focused on sensitive issues encountered at Holy water sites related to physical abuse, use of shackles to restrain patients, physical segregation and sense of alienation of patients with severe mental illness. The religious healers tried to justify these practices with multiple reasons, citing the necessity of using force when dealing with severely agitated patients during Holy water ceremonies in order to exorcize the evil spirits from their patients. Following the identified principles for a respectful, non-judgmental, mutual learning workshop, these discussions helped participants to gain understanding, perspective, and build trust and rapport. The workshop was facilitated through learning tasks, case scenarios, mutual sharing of experiences, and explorative critical questioning. Throughout the discussions, YB was also an empathetic listener and appraiser.

At the end of the first workshop, religious healer participants agreed to reflect on the discussions and further self-examine their understanding of mental illness; and to formally or informally share them with other church members who were not in attendance so they could discuss the new perspective learned from the workshop with their peers. Participants also promised to summarize the result of their discussions in a second workshop. Feedback from the religious healer showed that they wanted to learn more from mental health practitioners about how to handle aggressive mentally ill patients in subsequent workshops. All participants of the workshop agreed to have more consultative workshops and the responsibility was given to YB to plan the next session at a hospital.

### The second consultative workshop—exploration of new ideas and relationships

The second workshop included visits to the emergency department and outpatient clinics at a major hospital in Addis Ababa. In the clinic, the facilitator (YB) described and explained how the psychiatric system is organized. After the half day visit to the clinic, the workshop began as participants gathered in a hospital meeting room. There, the participants reviewed their reflections, thoughts and opinions that were raised from the first workshop; and the remaining time was dedicated to newer questions and ideas related to their observations during the hospital visit. The most frequently asked questions by religious healers were how do doctors manage physically agitated and aggressive patients? And how do doctors fight stigma and segregation of mentally ill patients? Religious healers shared that they were surprised and impressed after witnessing the effective way the hospital's emergency department managed aggressive and agitated patients, and started changing some of their old beliefs about the need to use force to manage similar patients at Holy water sites.

### The third and fourth consultative workshops—exploration of new relationships and roles and planning a course of action

The third and fourth workshops were conducted within the vicinity of the St. Michael's Church. The two workshops reviewed previous workshops' core topics and landing points and offered opportunities to begin exploring an alternative approach in handling mentally ill people at Holy water sites. The third workshop started by visiting a Holy water ceremony at St. Michael's Church, followed by a discussion continued in the church meeting room. During the visit, mental health practitioners witnessed beatings, arm twisting, forced bathing, shackling, and otherwise rough handling of patients with mental illness. As discussed in the preceding workshops, the mental health practitioners respectfully raised the issue of physical violence involved in patient management during Holy water treatments and suggested to work together to improve the processes. Some of the religious healers argued that to bring out evil spirits from the patients and properly bath severely agitated mentally ill patients, they needed to use force. Some religious healers in the group did share feelings of discomfort and were able to critically examine their taken-for-granted assumption that the use of shackles and force in severely agitated patients was necessary. After some discussion, the majority of religious healers accepted some of their past practices of shackling patients were potentially unnecessary, and these realizations opened up the possibility of incorporating some new approaches that they had observed in the hospital.

The fourth workshop explored the possibility and fundamentals of a collaborative approach between biomedical sciences and religious healing. Participants were asked in smaller groups to re-visit, evaluate and discuss their approach to treating the mentally ill at Holy water sites. In the final large group discussion, the participants articulated a desire to explore collaboration and improve care. During this process the facilitator (YB) of the workshop was not promoting any particular model, and intentionally created a space that was conducive to participants constructing their own version of collaboration. Participants of the workshop suggested an outpatient clinic to be established within the vicinity of the Holy water treatment communities. The mandate of the clinic would be to welcome any patient seeking Holy water treatment at the site who was also interested in receiving biomedical consultation and possible treatment. The workshop attendants accepted such a dual approach and the proposed collaboration plan was thoroughly discussed and accepted by the majority of the participants. Religious healers expressed their endorsement and willingness to refer their clients to the medical practitioners. However, they emphasized the need to use Holy water treatment at the same time.

## Results

In this collaborative endeavor, we identified evidence of transformative learning in both the biomedical practitioners and religious healers, consistent with most of the phases, but particularly phases 1, 2, 3, and 5 of Mezirow's framework (43) (see Table 1). While the data is not segregated, the majority of the study's participants were religious healers. As such, the transformative changes we describe represent in particular their experiences on the matter. In addition, our analysis revealed that changes was more profoundly reported by the religious healers.

**Table 1:** Mezirow's Phases of Transformation.

1. A disorienting dilemma
2. Self-examination with feelings of guilt or shame
3. A critical assessment of epistemic, sociocultural, or psychic assumptions
4. Recognition that one's discontent and the process of transformation are shared and that others have negotiated a similar change
5. Exploration of options for new roles, relationships, and actions
6. Planning a course of action
7. Acquiring knowledge and skills for implementing one's plans
8. Provisionally trying out new roles
9. Renegotiating relationships and negotiating new relationships
10. Building competence and self-confidence in new roles and relationships
11. A reintegration into one's life on the basis of conditions dictated by one's new perspective

To illustrate participant phases of transformation, we have identified and organized our results under four significant and recurrent themes related to the participants' experiences that contributed to their learning transformation. These are (1) Emotional distress—related to a disorienting dilemma; (2) Revisiting the etiological explanatory model—through self-examination and critical assessment of assumptions; (3) Working through a perplexing impasse—related to issues of physical and risk of sexual abuse, *via* recognition of discontent and eventual shared transformation; and (4) Exploration of new roles and relationships and willingness to take action- as advocates and collaborators to enhance service provisions for mentally ill patients. We will also explore the cultural adaptations encountered in applying Mezirow's framework of transformative learning.

### Emotional distress

The consultative workshops created an opportunity for workshop participants to face some common problems and assumptions. Different scenarios in the consultative workshop created emotional distress in some of the participants. For example, after visiting the Holy water sites one of the mental health practitioners reported: “*It is very sad to witness this, but many are forced to eat, sleep, urinate, defecate in a very small space, sometimes no larger than a one- to two-meter radius.”*

Similarly, a religious healer participant reported: “*For the last 20 years of my Holy water service experience, I encountered highly agitated patients, some of them were physically chained for months, others were seriously physically injured, and the remaining attempted to kill themselves by jumping from the cliff. It is painful and un-Christian to witness all these human suffering in our Holy water sites… we have to do something to change this…”* These emotionally charged distresses, in the forms of shock, guilt or shame were related to a disorienting experience after a new encounter, or dilemma when compelled to re-examine a routine practice. These distresses are pivotal in bringing forward further reflections and critical assessments as part of transformative learning.

### Revisiting the etiological explanatory model

The workshop was facilitated through learning tasks, case scenarios, mutual sharing of experiences, and explorative critical questioning and these activities created opportunities for some participants to revisit their etiological explanatory model and recognize the common denominators from both healing paradigms. For example, one of the religious healers reported: "*I was thinking the severely physically agitated patients in the hospital emergency room were attacked by evil spirit, and like the causation of the problem the solution was to force full Holy water treatment, not medications and what I used to believe was an injection would worsen the agitation and complicate the presence of evil possessions. However, when I saw the effectiveness of the injection to calm the patients without any physical restraint in a few minutes I was perplexed and started re-inquiring my previous perspective.”*

Another religious healer participant reported: “*I am puzzled and astonished in the first workshop by mental health practitioners' attribution of mental illness not to the usual and only biological causes but also to poverty, migration, war, abuses, alcohol, marijuana and khat. This means we have a lot in common.”* Along with emotional distress, the newly acquired knowledge and positive recognition of useful and shared perspectives also helped to promote critical assessment of previously held assumptions, paving further the road for transformative learning.

### Working through a perplexing impasse

One of the most challenging phases in the process of transformation for mental health practitioners as well as religious healers was discussing the issues of physical and sexual abuses of patients with severe mental illness around Holy water sites. One of the religious healers recounted: “*What I found, frankly, horrific is seeing severely mentally ill people shackled or locked in cage or sheep shed in overcrowded and unsanitary environment around the Holy water sites. Families are struggling to cope without any support from the church or government, it is really heartbreaking …”*

Another religious healer reported: “*One of my shackled patients with distress told me how the chain is so heavy, physically and mentally painful to walk around with chains at Holy water sites.”*

Drawing from Biblical knowledge, another religious healer emphasized: “The *Scripture says God created human beings. He created them godlike, reflecting God's nature. Genesis 1:27 said: mentally ill people are humans like us, they need maximum care from us; my church members agreed to do everything to stop physical abuse and segregation because it is our religious duty to exercise safety for all.”*

One mental health practitioner participant also reported his concern about the risk of sexual abuse: “*At Holy water sites the way they (patients) are left to wander in the forest. The female patients with severe mental illness can be impregnated. The baby born will be at risk of getting an unfriendly environment. That means two peoples become affected, the baby and the mother.”*

These accounts document the deep level of engagement and motivation of participants from different groups, aiming to chang ways of care that up until then were considered routine. Focusing on topics of patient mistreatment allowed participants from both groups to draw on their respective moral-ethical reasoning schemes to arrive to a shared goal of improving conditions of care.

### Exploration of new roles, relationships and willingness to take action

The tension created by disorienting dilemmas associated with the participants' usual beliefs, along with new insights from the consultative workshops, encouraged them to reassess their previously held perspectives, thereby pushing them to play new roles as advocates and collaborators for the good of mentally ill patients. For example, one religious healer participant reported: “*After the first workshop in my church we informally discussed about stigma and physical abuses of mentally ill people. Everyone shared this concern regarding the issue. Though the Scripture says God is near to the brokenhearted and saves the crushed in spirit (Psalm 34:18) we failed to fully exercise; we agreed to find a solution to curb the problem.”*

One mental health practitioner reported: “*We have no choice other than collaboration with religious healers as we share patients and share similar problems in serving people with mental illness; there is a lot to learn from the (religious) healers' communication skills and spiritual calmness while they interact with severely agitated patients.”*

Furthermore, after learning about it from the workshops, religious healer participants recommended that Christian communities around Holy water sites be educated about biopsychosocial aspects pertaining to mental illness. They believed that such education would be helpful in curbing the stigma experienced by the people with mental illness and their families. They anticipated that information from their updated and changed perspectives would also help and enable the community members to be more understanding of the situation. Participants asserted that this should be communicated clearly to communities, clergy men, and Holy water attendants so that they would stop treating people with severe mental illness negatively. Taken together, the religious healers have moved from facing dilemmas, moving through impasses, revaluating assumptions, to arrive at taking actual actions to create plans to improve practices and taking on new roles and relationships.

### Cultural adaptations in employing mezirow's transformative learning framework

Although Mezirow's phases of Transformative Learning was transferrable to the Ethiopian context, we found that some cultural adaptations were required for the workshops to be successful (see Table 1). Knowing that the learning is conducted across different local and professional and spiritual cultures, and being aware of differing attitudes, the main facilitator (YB) of the workshops paid particular attention to acknowledge and accommodate the target audiences' ways of knowing, value systems, and their understanding of reality. Because much of the new knowledge was communally and collaboratively produced in the workshops, it was more accepted and the further dissemination of knowledge to the rest of the community by religious healers through informal and formal discussions was notably easier. In the workshops, with a rich cultural backdrop, the way of learning and knowing and transmitting knowledge is heavily determined by contextual Ethiopian values. We outline some examples of how cultural values inform, guide and shape different aspects of Mezirow's transformational learning (see Table 2).

**Table 2:** Cultural adaptations to Mezirow's Phases of Transformation.

**Participants' and Organizers' report**	**Ethiopian values that inform the process**
“*Since the orthodox Christian church is highly hierarchical the letter from its headquarters proved to be useful as a way of introduction, gaining the trust of local spiritual leaders, and made the local priests practicing Holy water treatment much more comfortable and authorized to engage in such novel conversation with someone working outside their religious community.”*	Attentive to established hierarchy
“*To facilitate more acceptance and respect the religious tradition YB approached the headquarters of the Church in the capital (Addis Ababa) that is responsible for overseeing all church functions”*	Being respectful to social order and norm
“*With due respect to Holy water service the physical manipulation of patients with severe mental illness during holy water treatment has to change and suggested to work to gather to improve the processes of Holy water.”*	Being humble and not judgmental
“*At the end of the workshop participants agreed to reflect on workshop's discussions and to formally or informally share the workshop discussion with other church members so that they are able to do self-examination of their understanding of mental illness and share the new perspective from the workshop about mental illness.”*	Valuing community participation Continued learning Having humility
“*After the first workshop in my church we informally discussed about stigma and physical abuses of mentally ill people. Everyone shared his concern regarding the issue. Though the Scripture says God is near to the brokenhearted and saves the crushed in spirit (Psalm 34:18) we failed to fully exercise; we agreed to find a solution to curb the problem.”*	Valuing betterment of the collective
“*The Scripture says God created human beings. He created them godlike, reflecting God's nature. Genesis 1:27: mentally ill people are humans like us they need maximum care from us; my church members agreed to do everything to stop physical abuse and segregation because it is our religious duty to exercise safety for all”*.	Spirituality as service to people

## Discussion

The current study identified a number of self-reported accounts and observable actions that are indicative of transformative learning amongst all participants. To properly examine and evaluate these changes, TLT as articulated by Mezirow (12) has been a valuable framework that lends insight and understanding on the transformation that took place in the participants who were involved in the workshops that were part of a collaborative project between biomedical and traditional healers—practitioners who are normally widely apart in their paradigms of care. The Mezirow framework was also adaptable and relevant to this innovative adult learning project that is deeply cross-cultural.

Religious healers reported more profound transformative experiences than participating biomedical practitioners, particularly with regard to their experiences with a disorienting dilemma. This finding may be attributed to the fact that more religious healers participated than biomedical practitioners in the study. However, the findings may also be rooted in the Ethiopian cultural reality. Given that the majority of the population has access to traditional healing, there is likely a certain familiarity with traditional healing by the biomedical practitioners, while general access to biomedical perspectives, hospital sites and outpatient clinics are not as available or desirable by the religious healers. This could explain how and why they experienced more disorientation, distress, impasse, and dilemma, and that our findings slightly skew toward the transformation of healers rather than biomedical providers. Through careful and informed design, the consultative workshops created a participant-centered, respectful, and trusting environment, which, in turn, promoted a more genuine, interactive discourse. We believe this open, non-threatening setting to be a key factor that contributed to the success of these workshops, similar to other researchers who have paid particular attention to creating a safe place for learning, particularly in a cross-cultural setting, working with sensitive materials (46, 47). The importance of this in the current study could be inferred from how it allowed productive processing of the disorienting dilemmas and emotional distresses encountered by the participants. The workshops identified disorienting dilemmas in encountering forms of physical maltreatment of patients at the Holy water sites. The resulting emotions of shame, embarrassment, annoyance, or anger would have likely created an impasse that could have been hard to overcome had the safe space been absent, as other researchers have often discussed (47, 48).

The participants also took on risk by inviting each other to their home bases and exposing themselves in their thoughts and work in sensitive areas, with threats to pride, professional identity, and reputation. Education researchers have also identified the concept that to learn is to entertain risk, and learning takes one to the zone of discomfort and incompetence (49). The risk taking and learning occurring at the Holy water treatment sites and hospital visits exposed participants to uncomfortable practices and their counterparts' potentially more effective practices that may threaten their own ways of understanding. These exposures, however, guided by a thoughtful and respectful reflective debriefing, likely contributed to the transformative learning as the participants chose to reassess, rather than reject and withdraw from deliberating the things that caused them distress. This is consistent with research that examines the notion of “losing face” related to a “learning–credibility tension” where learners must overcome their sense of insecurity, with courage, and take risk to master new knowledge to aid the transformation (50). Furthermore, it is a credit to the religious healers that the spark for transformative learning is likely related to their desire to improve mental healthcare for distressed patients.

Beyond a “learning-credibility tension”—typically observed in newcomers to a learning setting, the religious healers' reflections and reassessments carried out in the workshops and beyond could not have been easy, when one considers their established high social status, and the well-entrenched and culturally enshrined practices. To address this dynamic, it is notable that the facilitator of the workshops purposively minimized hierarchical relationships between the biomedical and traditional healers, invited a religious healer to co-lead workshops, and maximized horizontal dialogues that equitably valued participation and reciprocity (51). A further consideration of power dynamics is related to the role of the facilitator itself. While power inequalities within the facilitator-participants' interaction cannot be eliminated—for instance socioeconomic, educational, and opportunity inequities—the facilitator strived to create a collegial and sharing environment that appeared to have contributed to a dynamic discourse among the participants. In addition, the exquisite attention to power dynamics is a common and significant factor for successful collaborative learning and to avoid resistance in general (52). Related to this attention to power dynamics in the workshops is also the importance of conducting learning activities with genuine respect and humility, and being aware of the vast professional and cultural diversity. Researchers have emphasized cultural humility through an anti-bias approach in transformational learning settings, and the current study highlights its importance (53).

Part of the reason for the workshops' ability to give rise to notable transformation learning may also be that the topics under discussion were interesting and the new knowledge was practical and useful, an important foundational factor in learning (54). The cross-professional and cross-cultural exchange is very innovative and unique by most conventional learning standard. The workshops conducted in the current study had no shortages of dilemmas and distressful emotions (13), that were likely instrumental in propelling the religious healers to reassess and reframe their assumptions about their practice, particularly around the management of severely agitated patients. The workshops helped them to develop another perspective as most expressed their dissatisfaction on this regard. Their discontent motivated the healers for critical evaluation of the situations and prepared them for change. Furthermore, paying particular attention to the emotional aspects, the theory of “edge-emotions” proposes that resistance to new learning and reflection may be rooted in cognitive functions and neurobiology of emotions that act to serve self-preservation (55). The theory also advocates that edge-emotions could be harnessed in learning to constructively support critical reflection and transformative learning. This harnessing of the emotional reactions of shame and urge to improve the physical handling of patients from the religious healers that led to reflection and action were exemplary in this regard.

The current study has a number of additional aspects that were likely conducive to transformative learning. For one, there was great attention paid to the premise that learning is both an individual and social process (43). The purposively promoted critical reflections at the workshops also occurred at both the individual and group/community levels. Using the uncomfortable and *status quo* challenging question of whether religious healers need to shackle the patients as an example, the transformational change could not have only taken place at an individual level as it is a collective cultural practice, but it had to start at the individual level with one's willingness and courage to reflect. More specifically, we observed in these workshops two types of learning discourses. The first is a formal discourse which occurred within the workshops among the individual participants. At this level, vibrant discourse for transformative learning may be best accomplished in a “group care” setting where more participants are present and a peer-to-peer conversation can develop (56), and the facilitator can more effectively fulfill extra roles in transmitting information, and promoting reflection and new behavior (57). The peer-to-peer aspects of learning seem to be evident in the current study.

Second is the desire of both the greater church-related community and the biomedical practitioners at the hospital to share information about mental illness—as exemplified by the group's desire and plans to disseminate psychoeducation to the community to decrease stigma related to mental illness, and to help the patients and their families. Although this type of informal discourse can be hard to develop in an official setting, the participants' own initiative and the facilitator's encouragement to the participants to share the information to other members of the church made this more possible. The workshops themselves were able to promote such individual and group/community level changes through producing new materials, and opportunities to assess and present valid evidence to support the new ways of thinking (38). Research has found that informal discourse may even have gender specific properties—for example, women may resonate with informal discourses more than formal didactic methods (58). In this case, the all-male established priest healers may still share a preference for this approach given the informality and freedom to choose and decide what is relevant and having group level blessing on the content. When successful, transformative learning such as this could lead to community and social level changes. Esperat and colleagues used transformational learning theory, as described by Freire (59), to develop a framework for participatory action research that were useful in combating childhood obesity (8, 60). Within participatory action research, people participate in research design to produce and implement interventions appropriate to their culture or local environment (61). This approach allows participants to identify their own disorienting dilemma, or, in Freire's terms, to achieve critical consciousness through interactions with the facilitator (8). Similar research on transformative learning were employed to address even larger societal issues such as racism and sexism (62, 63), and the current project's results holds promise for larger community and societal level changes in addressing stigma against mental illness, for example.

In later phases of Mezirow's TLT, regarding taking actions and trying on new roles and identities, the workshop achieved these key transformative learning steps in the participants' endorsing a new biomedical clinic and pledging to make referrals to the Clinic. As the workshop organizers had hoped, the result of the workshops went beyond providing information; rather, it shifted the role of religious healers to collaborators with mental health practitioners and advocates in area of mental health. Debate exists about whether transformative learning should lead to action. Freire, who influenced Mezirow's theories, believed that transformational learning will naturally lead learners to realize the presence of inequities and work to fix or overthrow unjust structures (13, 59, 64). However, Mezirow does not agree that transformative learning always leads to visible action. While he does agree that action is usually the last step in transformative learning, the action may be a decision rather than a measurable change in behavior (43). For the current study, it is very validating to report that these actions and new roles have been further fulfilled by the religious healers indeed as our recent related research on the clinic has confirmed (65).

Last, but not least, culturally informed considerations and adaptations were present throughout the workshops. In the entire process, all designs, communications, activities, and formal and informal content were carried out with active incorporation of Ethiopian cultural and religious values to facilitate successful transformative learning. In the workshop facilitation, the facilitators also consciously or subconsciously employed Ethiopian specific cultural and religious values to promote transformative learning. Having the ability to understand, respect and successfully deal with others from a different professional, social, healing, or linguistic cultures is critical (66). The current study testifies that tking into account the cultural context in which any learning takes place is crucial to achieving that learning. It would warrant further study to enhance this area as, to date, we were unable to locate any specific research which explicitly analyzed how culture shapes a transformative learning experience.

The current exploratory study has a number of limitations. Not unique to a focused ethnographic study is the lack of long term, in depth and disinterested data to evaluate the natural course of change, and the sustainability of the transformative learning observed here. The validation of the outcomes through triangulation is also limited. The pooled data studied how the whole body of participants underwent transformation but a resultant limitation is a lack of segregated data analysis to see how individual groups or members reacted or changed in particular. By nature of the collaborative project, there was no control group but having one would have been helpful to see if a “standard” approach would have been as effective. There is a large amount of work done by the experienced and knowledgeable research team that may be hard to operationalize or reproduce in other settings. The generalizability of the study may be limited as a result. Nevertheless, the principles and detailed descriptions of the content may help ameliorate this limitation, allowing for transferability of procedures for enabling stronger collaboration between traditional healers and biomedically trained practioners.

## Conclusion

Our study demonstrates the utility of the TLT in both the design and evaluation of initiatives aiming to bridge cross-cultural and cross-professional divides. The learning process was further enhanced by a collaborative participatory action model adjusted to accommodate Ethiopian socio-political and cultural relations. This innovative approach for using education as a vehicle for health system reform has broad transferability to other health sectors motivated to integrate biomedical models of care with alternative ways of knowing and caring. The implementation of collaboration strategy harnessed the complementarity of traditional and biomedical sectors of mental health care as a means of improving access to services. The successful interaction between the traditional and biomedical sectors, facilitated by TLT, has created a unique mental health service model warranting further study.

## Data availability statement

The raw data supporting the conclusions of this article will be made available by the authors, without undue reservation.

## Ethics statement

The studies involving human participants were reviewed and approved by Institutional Review Board of Addis Ababa University (AAUMF 01-008). The patients/participants provided their written informed consent to participate in this study.

## Author contributions

YB and DW had a major role in initiating the idea and constructing the conceptual framework of the study. YB and SL designed the study. YB was involved in data collection sessions, analysis, and manuscript writing. SL, SS, and MAM were involved in verification of the results and manuscript writing. All authors contributed to the article and approved the submitted version.

## Conflict of interest

The authors declare that the research was conducted in the absence of any commercial or financial relationships that could be construed as a potential conflict of interest.

## Publisher's note

All claims expressed in this article are solely those of the authors and do not necessarily represent those of their affiliated organizations, or those of the publisher, the editors and the reviewers. Any product that may be evaluated in this article, or claim that may be made by its manufacturer, is not guaranteed or endorsed by the publisher.
